# Second generation silver(I)-mediated imidazole base pairs

**DOI:** 10.3762/bjoc.10.221

**Published:** 2014-09-09

**Authors:** Susanne Hensel, Nicole Megger, Kristina Schweizer, Jens Müller

**Affiliations:** 1 Institut für Anorganische und Analytische Chemie, Westfälische Wilhelms-Universität Münster, Corrensstr. 28/30, 48149 Münster, Germany

**Keywords:** bioinorganic chemistry, DNA, imidazole, metal-mediated base pairs, nucleic acids, nucleosides

## Abstract

The imidazole–Ag(I)–imidazole base pair is one of the best-investigated artificial metal-mediated base pairs. We show here that its stability can be further improved by formally replacing the imidazole moiety by a 2-methylimidazole or 4-methylimidazole moiety. A comparison of the thermal stability of several double helices shows that the addition of one equivalent of Ag(I) leads to a 50% larger increase in the melting temperature when a DNA duplex with methylated imidazole nucleosides is applied. This significant effect can likely be attributed to a better steric shielding of the metal ion within the metal-mediated base pair.

## Introduction

Nucleic acids with their evolutionary optimized self-assembling properties represent an ideal basis for the generation of artificial, site-specifically functionalized supramolecular aggregates [1–4]. In this context, numerous functional moieties have been introduced into various nucleic acid building blocks, including, for example, modified nucleobases and modified sugar entities. A recent addition is the application of ligand systems as nucleobases surrogates [5–9]. In the presence of suitable transition metal ions, so-called metal-mediated base pairs between two complementary ligand-derived artificial nucleobases can be formed. Accordingly, metal-based functionality can be introduced site-specifically into nucleic acids. Various applications have been reported for such metal-modified nucleic acids [5], including increased charge transfer capability [10–11] and DNA-based logic gates [12–13]. Applying the concepts of coordination chemistry, a wide range of metal-specific ligands have been incorporated successfully into nucleic acids and nucleic acid derivatives. Most recently, even coordination patterns of the [2 + 1] type could be obtained [14–15]. While most examples focus on the use of DNA, other systems such as RNA [16], GNA [17–18] (glycol nucleic acid), PNA [19–21], and other nucleic acid derivatives [22] have been modified as well. It is interesting to note that metal-mediated base pairs do not necessarily require the presence of artificial ligands. In contrast, natural nucleobases have also been successfully applied in the generation of metal-mediated base pairs such as thyminate–Hg(II)–thyminate or cytosine–Ag(I)–cytosine [23–25]. It has even been shown that polymerases are capable of processing metal-mediated base pairs [26–27], resulting, e.g., in the formation of cytosine–Ag(I)–adenine base pairs [28]. Structural analyses of short oligonucleotide duplexes comprising one or more metal-mediated base pairs indicate that the large conformational space of nucleic acids exists also for metal-modified nucleic acids. In particular, the existence of B-DNA- and Z-DNA-type conformations have been reported in this context [29–33].

**Scheme 1:**
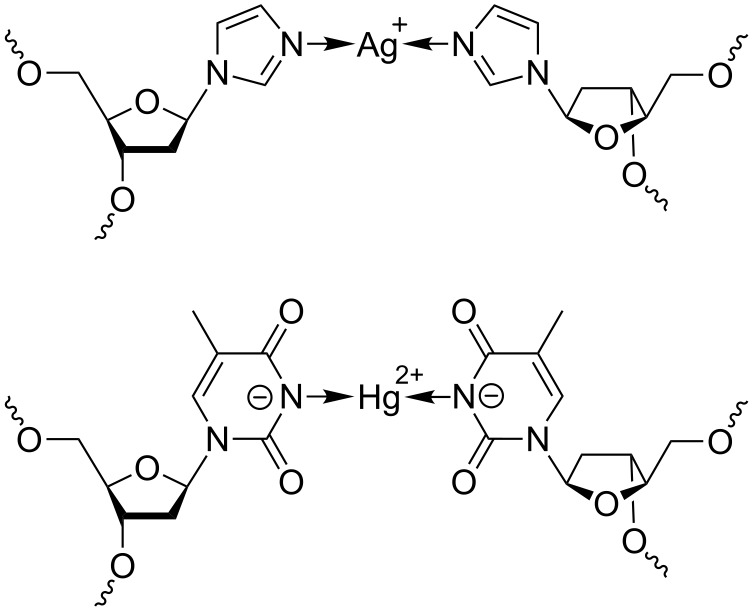
Schematic representation of an imidazole–Ag(I)–imidazole base pair (top) and a thyminate–Hg(II)–thyminate base pair (bottom).

**Figure 1:**
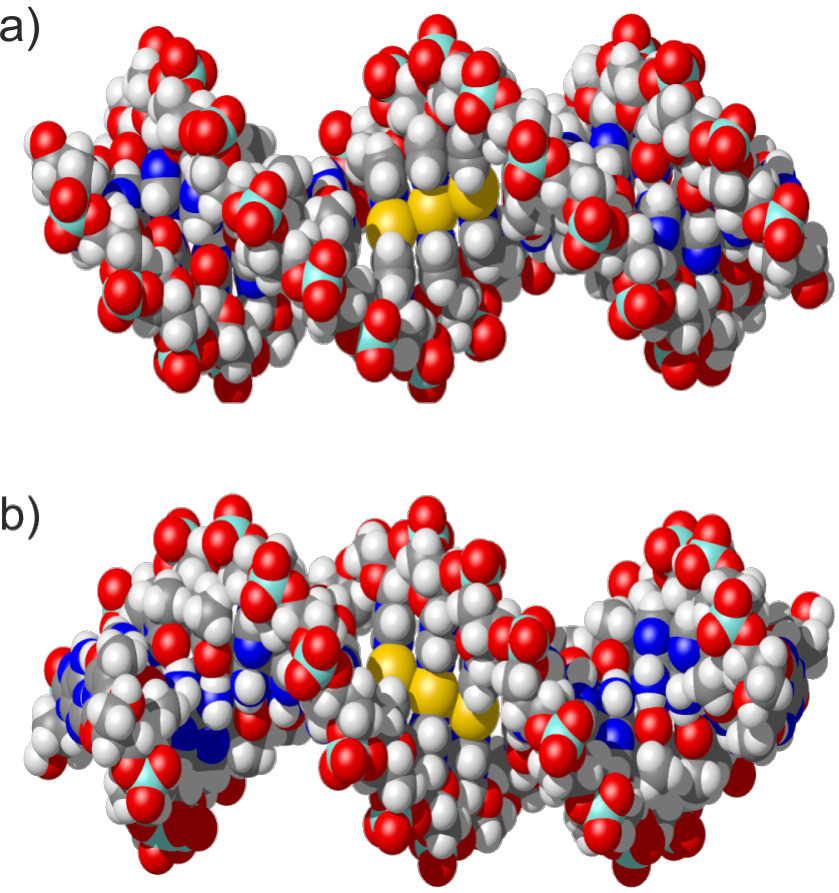
Different views of a DNA duplex comprising three neighbouring imidazole–Ag(I)–imidazole base pairs. a) View from the major groove; b) view from the minor groove. The silver(I) ions shown in yellow are clearly visible and appear to be solvent accessible. Coordinates have been taken from PDB 2M54. This figure was prepared using MolMol [37].

The imidazole–Ag(I)–imidazole base pair represents one of the best investigated metal-mediated base pairs to date [29–30,34–35]. It comprises a linearly coordinated silver(I) ion inserted in-between two complementary artificial imidazole nucleosides (Scheme 1, top). The NMR-based solution structure of a DNA duplex with three contiguous imidazole–Ag(I)–imidazole represented the first experimental proof that a B-DNA conformation is compatible with the presence of metal-mediated base pairs, as only minor distortions at the base pair steps between natural and artificial base pairs were observed [29–30]. Additional experiments have shown that neighbouring imidazole–Ag(I)–imidazole base pairs are formed cooperatively, suggesting the possibility of creating long contiguous metal-containing sections within oligonucleotide duplexes [35]. A closer inspection of the experimental NMR structure reveals that the silver(I) ions, despite being lined up inside the duplex along the helical axis, still appear to be accessible from the outside of the duplex (Figure 1). This contrasts the situation in a duplex with thyminate–Hg(II)–thyminate base pairs (Scheme 1, bottom) [31–32]. As a result of the keto groups on the thymine residues, the mercury(II) ions included in this type of metal-mediated base pair are clearly shielded from outside access [31]. In an attempt to increase the stability of the imidazole–Ag(I)–imidazole base pairs, additional shielding of the central silver(I) ions could be accomplished by introducing methyl groups on the imidazole moieties. A comparison of the p*K*_a_ values [36] of 1-methylimidazole (p*K*_a_ = 7.21), 1,2-dimethylimidazole (p*K*_a_ = 8.22) and 1,4-dimethylimidazole (p*K*_a_ = 7.75) indicates that this modification is also expected to slightly increase the basicity of the ligand, thereby also contributing to the formation of a more stable metal complex.

## Results and Discussion

### Synthesis and characterization of the nucleosides

**Scheme 2:**
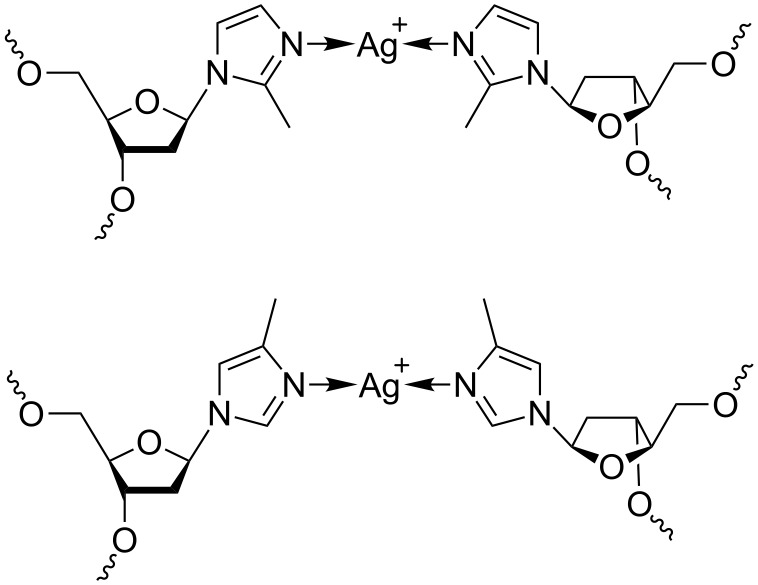
Schematic representation of silver(I)-mediated imidazole homo base pairs involving 2-methylimidazole (top) and 4-methylimidazole (bottom), designed to shield access to the silver(I) ions from minor and major groove, respectively.

**Scheme 3:**
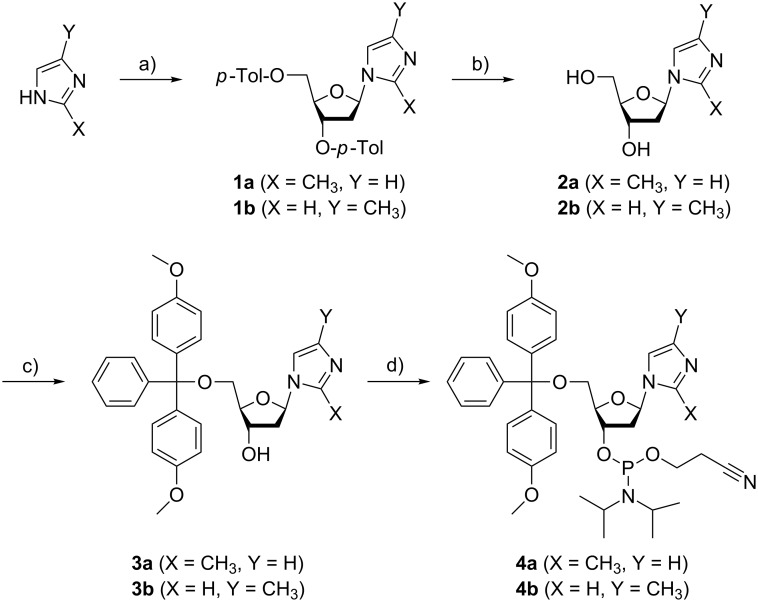
Synthesis of methylimidazole-based nucleosides and their corresponding phosphoramidites required for automated solid-phase DNA synthesis. a) 1. NaH, 2. Hoffer’s chloro sugar, CH_3_CN, 0 °C to ambient temperature, 3 h; b) aqueous NH_3_ (25%), CH_3_OH, ambient temperature, 16 h; c) dimethoxytrityl chloride, 4-dimethylaminopyridine, pyridine, ambient temperature, 3 h; d) 2-cyanoethyl-*N*,*N*-diisopropylchlorophosphoramidite, diisopropylethylamine, CH_2_Cl_2_, ambient temperature, 30 min.

Scheme 2 shows silver(I)-mediated methylimidazole homo base pairs involving either 2-methylimidazole (top) or 4-methylimidazole (bottom), resulting in a shielded access to the silver(I) ions from the minor and major groove, respectively. The phosphoramidites required for automated DNA solid-phase synthesis were obtained using synthetic procedures established previously for the non-methylated imidazole analogue (Scheme 3) [34,38]. Methylimidazole, deprotonated in situ via the addition of sodium hydride, has been reacted with Hoffer’s chloro sugar to give the *p*-toluoyl-protected nucleoside (**1a**, **1b**). In the case of 2-methylimidazole, only one product (2-methylimidazole nucleoside, **1a**) was formed due to the symmetry of the deprotonated starting compound. When performing this reaction with 4-methylimidazole, two product isomers were obtained as a result of the asymmetric substitution of the deprotonated starting compound, namely 4-methylimidazole nucleoside (**1b**) and 5-methylimidazole nucleoside. The latter isomer was obtained in very low yield only (isomer ratio of ~15:1). It was not further investigated, because its methyl group is oriented in a direction that is not expected to lead to an increased shielding of the silver(I) ions in the corresponding metal-mediated base pairs. As a result, all further experiments were performed using either **1a** or **1b** only. Deprotection of **1a**/**1b** by means of aqueous ammonia in methanol led to the free nucleosides **2a**/**2b**. The next two steps involved the orthogonal protection of the two hydroxy functions with the dimethoxytrityl and the 2-cyanoethyl-*N*,*N*-diisopropylphosphoramidite moieties, finally resulting in the formation of **4a**/**4b** suitable for automated solid-phase oligonucleotide synthesis.

The p*K*_a_ values of the free nucleosides **2a**/**2b** were determined by pD-dependent ^1^H NMR spectroscopy. They amount to 6.61 and 6.50, respectively. As expected, the methylimidazole nucleosides are slightly more basic than the corresponding non-methylated parent nucleoside (p*K*_a_ = 6.01) [34].

### Investigation of the oligonucleotides

**Scheme 4:**
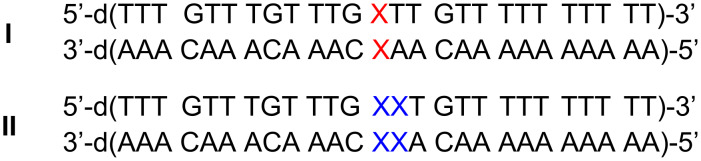
Oligonucleotide sequences under investigation (X = 2-methylimidazole **2a** or 4-methylimidazole **2b**).

**Figure 2:**
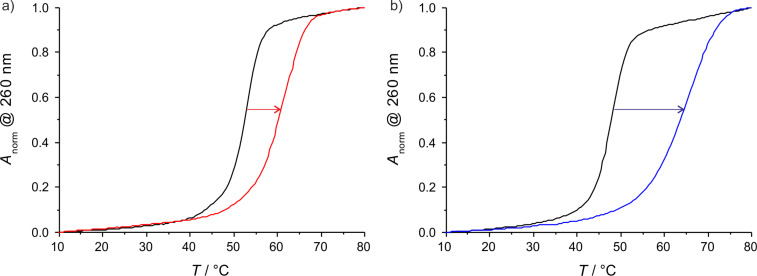
Melting curves based on normalized UV absorbance at 260 nm of a) duplex **I** with X = 4-methylimidazole and b) duplex **II** with X = 2-methylimidazole in the absence (black) and presence of one equivalent of Ag(I) (coloured). For the sequences, see Scheme 4. Experimental conditions: 1 μM duplex, 150 mM NaClO_4_, 5 mM MOPS (pH 6.8).

For each of the artificial nucleosides, two DNA double helices were synthesized comprising either one or two central methylimidazole:methylimidazole base pairs (Scheme 4). The sequences were chosen because they match those that had thoroughly been investigated previously with non-methylated imidazole nucleosides [35], enabling a direct observation of the expected shielding effect. Figure 2 shows the melting curves of two of the double helices, determined by temperature-dependent UV spectroscopy (for the other two duplexes, see Supporting Information File 1). As can clearly be seen, the addition of one equivalent [39] of Ag(I) leads to a significant increase in the melting temperature *T*_m_. Table 1 lists the experimentally determined melting temperatures. As expected, the double helix comprising two neighbouring methylimidazole:methylimidazole mispairs displays a lower *T*_m_ compared to the duplex comprising one mispair only. It is interesting to note that even in the absence of Ag(I) the duplexes containing the methylated imidazole nucleosides melt at a significantly higher *T*_m_ (by about 3–4 °C), possibly due to an additional stabilising hydrophobic interaction in the groove. The position of the methyl group (2-methylimidazole vs 4-methylimidazole) does not influence the absolute melting temperature. In the presence of one equivalent of Ag(I), the thermal stability of the duplexes increases significantly. Notably, the increase in *T*_m_ is larger by about 50% for the methylated imidazole nucleosides compared with the non-methylated one, amounting to about 8.5 °C per silver(I)-mediated base pair. The significantly increased thermal stability of the duplexes comprising either 2-methylimidazole or 4-methylimidazole therefore cannot be attributed to the above-mentioned hydrophobic interaction only but must also be a direct effect of the formation of the metal-mediated base pairs. This indicates that the concept of increasing the stability of silver(I)-mediated imidazole base pairs by restricting the access to the central metal ion via introduction of additional methyl groups on the ligand is successful.

**Table 1:** Absolute and relative melting temperatures *T*_m_ (°C) of DNA duplexes containing one or two (methyl)imidazole:(methyl)imidazole base pairs in the absence and presence of silver(I) (**Im** = imidazole; **2** = 2-methylimidazole; **4** = 4-methylimidazole; for sequences, see Scheme 4). The estimated standard deviation of *T*_m_ amounts to 1 °C. Experimental conditions: 1 μM duplex, 150 mM NaClO_4_, 5 mM MOPS (pH 6.8).

artificial nucleoside X	*T*_m_ (no Ag(I))	*T*_m_ (+ Ag(I))	Δ*T*_m_ (0 → 1 equiv Ag(I))

**Im**:**Im**^a^	49.5	55.5	+ 6.0
**2**:**2**	53.0	62.0	+ 9.0
**4**:**4**	53.3	61.3	+ 8.0
**ImIm**:**ImIm**^a^	44.5	55.5	+ 11.0
**22**:**22**	48.4	64.9	+ 16.5
**44**:**44**	48.2	65.7	+ 17.5

^a^Data taken from [35].

**Figure 3:**
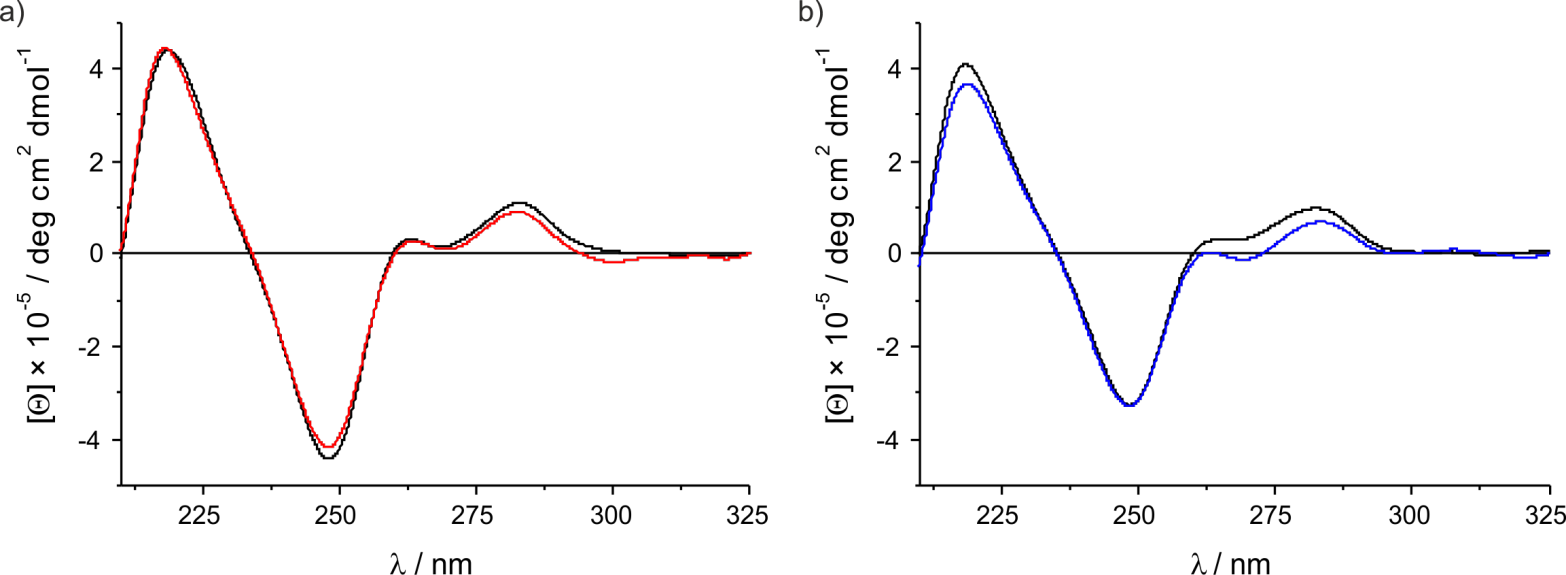
CD spectra of a) duplex **I** with X = 4-methylimidazole and b) duplex **II** with X = 2-methylimidazole in the absence (black) and presence of one equivalent of Ag(I) (coloured). For the sequences, see Scheme 4. Experimental conditions: 1 μM duplex, 150 mM NaClO_4_, 5 mM MOPS (pH 6.8).

The CD spectra shown in Figure 3 (and Supporting Information File 1) are in excellent agreement with those recorded previously for the analogous duplexes containing the non-methylated imidazole nucleoside. They indicate that the duplexes belong to the B-DNA family. The low intensity of the positive Cotton effects at about 262 nm and 283 nm is typical for duplexes with this type of sequence and has, for example, also been reported for poly[d(A)]·poly[d(T)] [40]. The presence of Ag(I) does not alter the wavelengths of the Cotton effects and only marginally affects the ellipticity at a given wavelength. Taken together, it can be concluded that neither the introduction of a methyl group nor the formation of the silver(I)-mediated base pair does influence the overall DNA duplex conformation. As the synthesis of 2-methylimidazole nucleoside from 2-methylimidazole yields one isomer only whereas the synthesis of 4-methylimidazole nucleoside from 4-methylimidazole also gives 5-methylimidazole as an undesired side product, it is recommended to replace imidazole by 2-methylimidazole in future research on imidazole–Ag(I)–imidazole base pairs to obtain nucleic acid systems of superior stability with otherwise identical properties.

## Conclusion

The formal substitution of an imidazole moiety in DNA duplexes comprising silver(I)-mediated imidazole–Ag(I)–imidazole base pairs by a 2-methylimidazole or 4-methylimidazole moiety leads to a significant increase in thermal stability of the nucleic acid. The increase in *T*_m_ upon the addition of one equivalent of Ag(I) rises by about 50% for the methylated nucleosides compared to the non-methylated one. The molecular structure of a DNA duplex with imidazole–Ag(I)–imidazole base pairs supports the notion that the increased stability may be due to a better shielding of the Ag(I) from the surrounding solvent.

## Supporting Information

UV melting curves and CD spectra of duplex **I** with X = 2-methylimidazole and of duplex **II** with X = 4-methylimidazole. Full experimental details and characterization data of the compounds shown in Scheme 3 and Scheme 4.

File 1 Experimental data.
