# Supplementary material for: Second generation silver(I)-mediated imidazole base pairs
Source: Beilstein J Org Chem. 2014 Sep 9;10:2139–44. doi: 10.3762/bjoc.10.221 (PMC4168905; doi:10.3762/bjoc.10.221)
Supplement: File 1 — Experimental data. [file Beilstein_J_Org_Chem-10-2139-s001.pdf]

Supporting Information

for

Second generation silver(I)-mediated imidazole base pairs

Susanne Hensel, Nicole Megger, Kristina Schweizer and Jens Müller*

Address: Institut für Anorganische und Analytische Chemie, Westfälische Wilhelms-Universität Münster, Corrensstr. 28/30, 48149 Münster, Germany

Email: Jens Müller - mueller.j@uni-muenster.de

* Corresponding author

Experimental data

Additional spectra

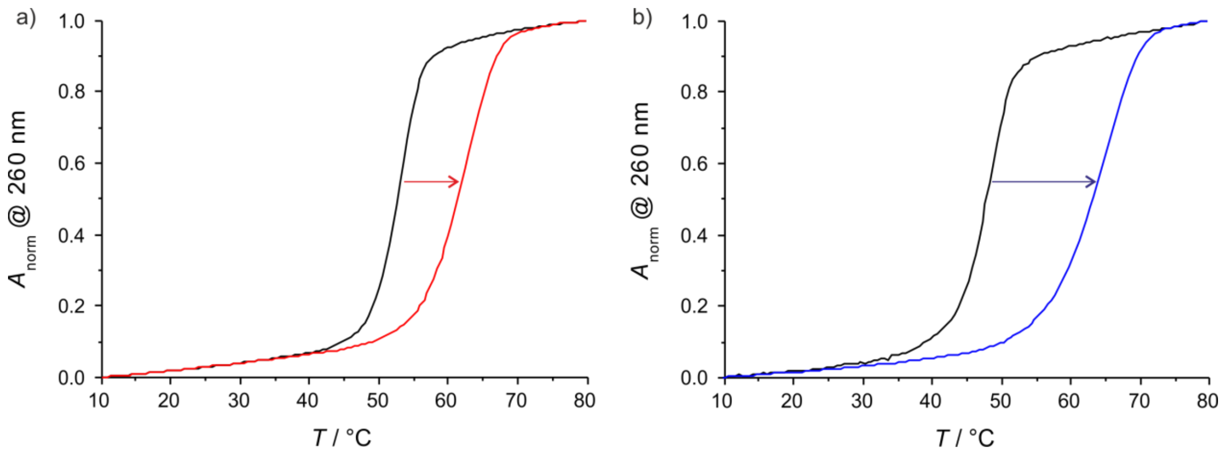

Figure S1: Melting curves based on normalized UV absorbance at 260 nm of a) duplex **I** with X = 2-methylimidazole and b) duplex **II** with X = 4-methylimidazole in the absence (black) and presence of one equivalent of Ag(I) (coloured). For the sequences, see Scheme 4. Experimental conditions: 1 μM duplex, 150 mM $NaClO_4$, 5 mM MOPS (pH 6.8).

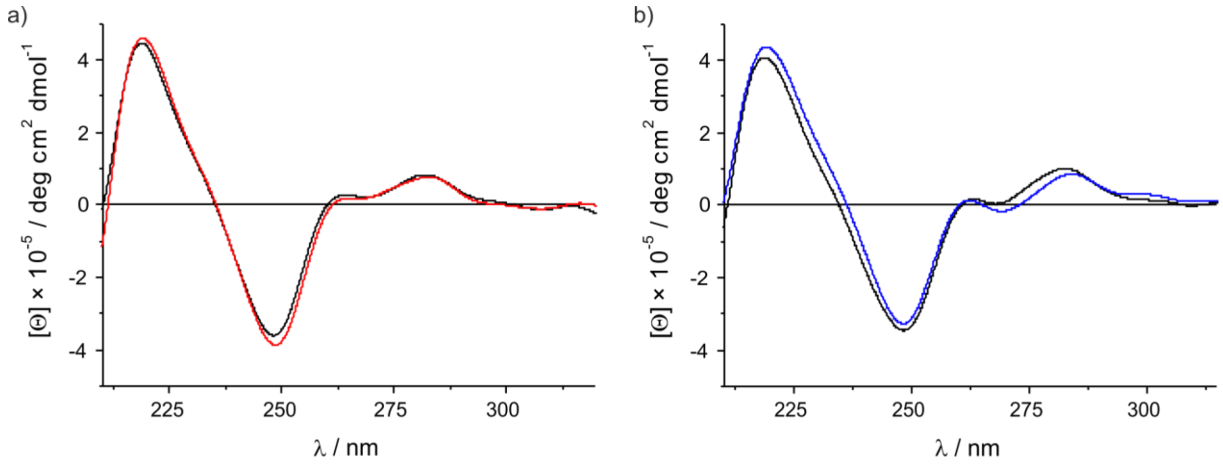

Figure S2: CD spectra of a) duplex **I** with X = 2-methylimidazole and b) duplex **II** with X = 4-methylimidazole in the absence (black) and presence of one equivalent of Ag(I) (coloured). For the sequences, see Scheme 4. Experimental conditions: 1 μM duplex, 150 mM $NaClO_4$, 5 mM MOPS (pH 6.8).

Experimental

DNA syntheses were performed in the DMT-off mode on a K&A Laborgeräte H8 DNA/RNA synthesizer by following standard protocols. The oligonucleotides were identified by MALDI-TOF mass spectrometry. MALDI-TOF mass spectra were recorded on a Bruker Reflex IV instrument using a 3-hydroxypicolinic acid/ammonium citrate matrix (5 mg 3-hydroxypicolinic acid, 12.5 µL ammonium citrate (50 mg/mL), 100 µL dist. Water, 100 µL acetonitrile) and applying a commercially available oligonucleotide with a molecular mass of 4590 Da as internal reference. NMR spectra were recorded using Bruker Avance(I) 400 and Bruker Avance(III) 400 spectrometers at 300 K. Chemical shifts were referenced to residual TSP (D_2O, δ = 0 ppm), TMS ($CDCl_3$, δ = 0 ppm), or to external H_3PO_4 (^{31}P NMR, δ = 0 ppm). UV/Vis spectra were recorded on a Varian CARY BIO 100 spectrophotometer in 1 cm quartz cuvettes with 1 µM duplex concentration. The melting profiles were measured in buffer (150 mM $NaClO_4$, 5 mM MOPS, pH 6.8). Temperature-dependent UV spectra were recorded between 10 and 80 °C with a heating/cooling rate of 1 °C min^{-1} and a data interval of 1 °C. Absorbance was normalized according to $A_{norm} = (A-A_{min})/(A_{max}-A_{min})$ at 260 nm. Melting temperatures have been determined as the maximum of the derivative of the annealing curves. CD spectra were measured with a JASCO J-815 spectrapolarimeter at 10 °C with intervals of 0.1 nm and a scan rate of 100 nm min^{-1}. 2-Deoxy-3,5-di-*O*-(*p*-toluoyl)-α-D-erythro-pentofuranosyl chloride was prepared according to a literature procedure.[1]

All reagents used were obtained from commercial sources and were used without further purification. The CH_2Cl_2 used in the synthesis of the phosphoramidites was dried over CaH. The products were purified by silica gel column chromatography (grain size 0.035–0.070).

Synthesis of **1a/1b**

The respective methylimidazole (385 mg, 4.70 mmol) was dissolved in acetonitrile (30 mL) and cooled to 0 °C. NaH (60% in mineral oil, 271 mg, 5.64 mmol, 1.2 equiv.) was added to the solution and the suspension was stirred at 0 °C for 30 min. After adding Hoffer´s chloro sugar (2.19 g, 5.64 mmol, 1.2 equiv.) in 4 portions during 60 min the reaction was stirred for further 3 h and allowed to reach ambient temperature. The solvent was removed under reduced pressure and the residue was dissolved in CH_2Cl_2. The organic layer was washed with water (3 × 30 mL) and dried ($MgSO_4$). The crude product was purified by column chromatography on silica gel (cyclohexane : CH_2Cl_2 : NEt_3, 50 : 30 : 8).

1´-(2-Methylimidazol-1-yl)-2´-deoxy-3,5´-di-*O*-*p*-toluoyl-β-D-ribofuranose (**1a**)

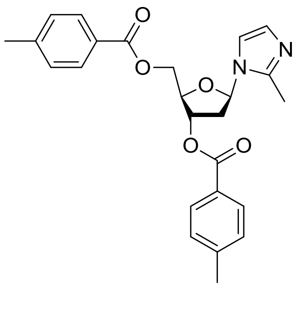

1a

White solid; yield: (1.02 g, 50%); ^1H NMR (400 MHz, $CDCl_3$) δ/ppm: 7.93 (m, 4H, Tol), 7.26 (m, 4H, Tol), 7.00 (s, 1H, H4), 6.91 (s, 1H, H5), 6.07 (pt, 1H, H1´), 5.65 (m, 1H, H3´), 4.60 (m, 2H, H5´/H5´´), 4.53 (m, 1H, H4´), 2.63 (m, 2H, H2´/H2´´), 2.48 (s, 3H, Tol-CH_3), 2.43 (s, 3H, Tol-CH_3), 2.41 (s, 3H, Im-CH_3); ^{13}C NMR (101 MHz, $CDCl_3$) δ/ppm: 166.2 (CO), 165.9 (CO), 144.8 (Tol), 144.6 (Tol), 144.2 (Tol), 129.7 (Tol), 129.3 (C4), 127.9 (Tol), 126.8 (Tol), 126.4 (Tol), 115.2 (C5), 84.8 (C1´), 82.2 (C4´), 74.9 (C3´), 64.1 (C5´), 38.9 (C2´), 21.8 (Tol-CH_3), 21.7 (Tol-CH_3), 13.4 (Im-CH_3); ESI-MS: m/z $(M+H)^+$ calcd = 435.19, found 435.19; Anal. Calcd for $C_{25}H_{26}N_2O_5$ × H_2O: C, 66.63; H, 6.24; N, 6.19; found: C, 66.57; H, 6.29; N, 6.13.

1´-(4-Methylimidazol-1-yl)-2´-deoxy-3,5´-di-*O*-*p*-toluoyl-β-D-ribofuranose (**1b**)

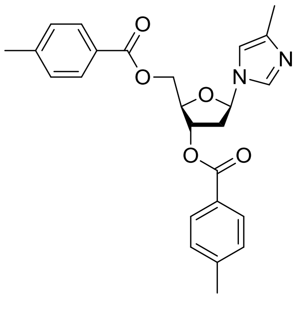

1b

Yellow oil; yield (595 mg, 29%); ^1H NMR (400 MHz, $CDCl_3$) δ/ppm: 7.93 (m, 4H, Tol), 7.59 (s, 1H, H2), 7.26 (m, 4H, Tol), 6.75 (s, 1H, H5), 6.06 (pt, 1H, H1´), 5.65 (m, 1H, H3´), 4.59 (m, 2H, H5´/H5´´), 4.54 (m, 1H, H4´), 2.65 (m, 2H, H2´/H2´´), 2.43 (s, 3H, Tol-CH_3), 2.41 (s, 3H, Tol-CH_3), 2.15 (s, 3H, Im-CH_3); ^{13}C NMR (101 MHz, $CDCl_3$) δ/ppm: 166.2 (CO), 165.9 (CO), 144.5 (Tol), 144.2 (Tol), 139.3 (Tol), 135.2 (Tol), 129.7 (Tol), 129.3 (C4), 126.8 (Tol), 126.4 (Tol), 112.6 (C5), 86.1 (C1´), 82.4 (C4´), 64.1

(C5´), 39.1 (C2´), 21.7 (Tol-CH_3), 21.6 (Tol-CH_3), 13.6 (Im-CH_3); ESI-MS: m/z $(M+H)^+$ calcd = 435.19, found 435.19; Anal. Calcd for $C_{25}H_{26}N_2O_5$ × 0.67 H_2O: C, 67.25; H, 6.17; N, 6.27; found: C, 67.30; H, 6.22; N, 6.17.

Synthesis of **2a**/**2b**

The respective *p*-toluoyl-protected nucleoside was dissolved in methanol (50 mL) and aqueous ammonia (25%, 25 mL) was added. The reaction mixture was stirred at ambient temperature overnight. The solvent was removed under reduced pressure and the crude material was purified by column chromatography on silica gel (CH_2Cl_2 : EtOAc : MeOH, 7 : 3 : 3).

1´-(2-Methylimidazol-1-yl)-2´-deoxy-β-D-ribofuranose (**2a**)

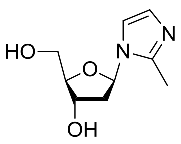

2a

White solid; yield: (373 mg, 80%); ^1H NMR (400 MHz, D_2O, pD 8.6) δ/ppm: 7.29 (s, 1H, H4), 6.97 (s, 1H, H5), 6.20 (pt, 1H, H1´), 4.56 (m, 1H, H3´), 4.07 (m, 1H, H4´), 3.75 (m, 2H, H5´/H5´´), 2.53 (m, 2H, H2´/H2´´), 2.46 (s, 3H, CH_3); ^{13}C NMR (101 MHz, D_2O, pD 8.6) δ/ppm: 146.5 (C2), 126.2 (C4), 116.5 (C5), 86.5 (C4´), 84.3 (C1´), 71.0 (C3´), 61.5 (C5´), 39.2 (C2´), 11.8 (CH_3); ESI-MS: m/z $(M+H)^+$ calcd = 199.11, found 199.11; Anal. Calcd for $C_9H_{14}N_2O_3$ × 0.25 H_2O: C, 53.32; H, 7.21; N, 13.83; found: C, 53.40; H, 6.91; N, 13.54.

1´-(4-Methylimidazol-1-yl)-2´-deoxy-β-D-ribofuranose (**2b**)

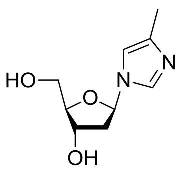

2b

White solid; yield (192 mg, 71%); ^1H NMR (400 MHz, D_2O, pD 8.8) δ/ppm: 7.81 (s, 1H, H2), 7.07 (s, 1H, H5), 6.13 (pt, 1H, H1´), 4.53 (m, 1H, H3´), 4.07 (m, 1H, H4´), 3.74 (m, 2H, H5´/H5´´), 2.53 (m, 2H, H2´/H2´´), 2.19 (s, 3H, CH_3); ^{13}C NMR (101 MHz, D_2O, pD 8.8) δ/ppm: 138.3 (C4), 136.5 (C2), 113.7 (C5), 86.6 (C4´), 85.8 (C1´), 71.0 (C3´), 61.6 (C5´), 39.6 (C2´), 12.1 (CH_3); ESI-MS: m/z $(M+H)^+$ calcd = 199.11, found 199.11.

Synthesis of **3a**/**3b**

The respective nucleoside (373 mg, 1.88 mmol) was co-evaporated with dry pyridine (2 × 10 mL). After dissolving in dry pyridine (8 mL) under argon, 4,4´-dimethoxytrityl chloride (1.2 equiv.) and catalytic amounts of dimethylaminopyridine were added and the reaction mixture was stirred for 3 h. CH_2Cl_2 (100 mL) was added and the organic layer was washed with sat. $NaHCO_3$ solution (3 × 30 mL). The crude material was purified by column chromatography on silica gel (CH_2Cl_2 : MeOH, 100 : 1 → 95 : 5).

1´-(2-Methylimidazol-1-yl)-2´-deoxy-5´-*O*-(4,4´-dimethoxytriphenylmethyl)-β-D-ribofuranose (**3a**)

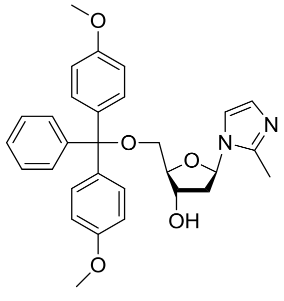

3a

Yellowish oil; yield: (760 mg, 81%); ^{1}H NMR (400 MHz, $CDCl_3$) δ/ppm: 7.40 (m, 2H, DMT), 7.25 (m, 7H, DMT), 6.94 (s, 1H, H4), 6.81 (s, 1H, H5), 6.80 (m, 4H, DMT), 5.99 (pt, 1H, H1´), 4.53 (m, 1H, H3´), 4.06 (m, 1H, H4´), 3.77 (d, 6H, OCH_3), 3.31 (m, 2H, H5´/H5´´), 2.42 (s, 3H, CH_3), 2.37 (m, 2H, H2´/H2´´); ^{13}C NMR (101 MHz, $CDCl_3$) δ/ppm: 158.6 (DMT), 144.6 (DMT), 135.7 (C4), 135.6 (DMT), 130.1 (DMT), 128.2 (DMT), 127.9 (DMT), 127.0 (DMT), 115.7 (C5), 113.2 (DMT), 86.6 (DMT), 85.8 (C4´), 84.5 (C1´), 77.4 (DMT), 76.8 (DMT), 72.3 (C3´), 63.9 (C5´), 55.2 (OCH_3), 41.1 (C2´), 13.2 (CH_3); ESI-MS: m/z $(M+H)^+$ calcd = 501.23, found 501.24; Anal. Calcd for $C_{30}H_{32}N_2O_5$ × 1.75 H_2O: C, 67.72; H, 6.72; N, 5.26; found: C, 67.63; H, 6.25; N, 5.30.

1´-(4-Methylimidazol-1-yl)-2´-deoxy-5´-*O*-(4,4´-dimethoxytriphenylmethyl)-β-D-ribofuranose (**3b**)

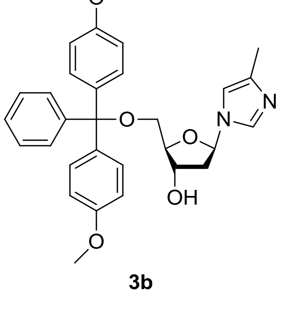

3b

Yellowish oil; yield (192 mg, 62%); ^{1}H NMR (400 MHz, $CDCl_3$) δ/ppm: 7.53 (s, 1H, H2) 7.40 (d, 2H, DMT), 7.26 (m, 7H, DMT), 6.82 (d, 4H, DMT), 6.71 (s, 1H, H5), 5.93 (pt, 1H, H1´), 4.50 (m, 1H, H3´), 4.04 (m, 1H, H4´), 3.78 (s, 6H, OCH_3), 3.31 (m, 2H, H5´/H5´´), 2.38 (m, 2H, H2´/H2´´), 2.15 (s, 3H, CH_3); ^{13}C NMR (101 MHz, $CDCl_3$) δ/ppm: 158.6 (DMT), 144.5 (DMT), 138.5 (C4), 135.7 (DMT), 135.7 (DMT), 135.0 (C2), 129.8 (DMT), 128.9 (DMT), 127.9 (DMT), 127.0 (DMT), 126.9 (DMT), 113.1 (DMT), 113.0 (C5), 85.9 (DMT), 85.8 (C4´), 84.8 (C1´), 72.2 (C3´), 64.0 (C5´), 55.2 (OCH_3), 41.3 (C2´),

13.4 (CH_3); ESI-MS: m/z $(M+H)^+$ calcd = 501.24, found 501.24; Anal. Calcd for $C_{30}H_{32}N_2O_5 \times 1.33\ H_2O$: C, 68.68; H, 6.66; N, 5.34; found: C, 68.85; H, 6.23; N, 5.09.

Synthesis of **4a**/**4b**

The respective DMT-protected nucleoside was dissolved in dry CH_2Cl_2 (15 mL) under argon atmosphere. *N*,*N*-Diisopropylethylamine (272 µL, 1.60 mmol, 4 equiv.) and 2-cyanoethyl-*N*,*N*-diisopropylchlorophosphoramidite (143 µL, 599 µmol, 1.2 equiv.) were added and the solution was stirred at ambient temperature for 30 min. Ethyl acetate was added (50 mL), the organic layer was washed with sat. $NaHCO_3$ solution (3 × 30 mL) and dried ($MgSO_4$). The crude product was purified by column chromatography on silica gel (CH_2Cl_2 : EtOAc : NEt_3, 75 : 23 : 2).

1´-(2-Methylimidazol-1-yl)-2´-deoxy-3´-((2-cyanoethyl)-*N,N*-diisopropylphosphoramidite)-5´-*O*-4,4´-dimethoxytriphenylmethyl-β-D-ribofuranose (**4a**)

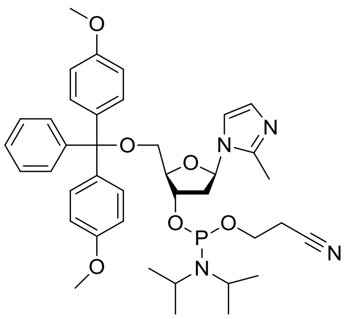

4a

Colourless oil; yield (132 mg, 47%) ^{1}H NMR (400 MHz, $CDCl_3$) δ/ppm: 7.41 (m, 2H, DMT), 7.27 (m, 7H, DMT), 6.96 (s, 1H, H4), 6.82 (s, 1H, H5), 6.80 (m, 4H, DMT), 5.99 (pt, 1H, H1´), 4.63 (m, 1H, H3´), 4.20 (m, 1H, H4´), 3.79 (s, 6H, OCH_3), 3.68 (m, 4H, CH_2), 3.61 (m, 2H, ^{i}Pr-CH), 3.29 (m, 2H, H5´/H5´´), 2.46 (m, 3H, Im-CH_3), 2.42 (m, 2H, H2´/H2´´), 1.18 (m, 12H, ^{i}Pr-CH_3); ^{13}C NMR (101 MHz, $CDCl_3$) δ/ppm: 158.6 (DMT), 144.6 (DMT), 135.7 (C4), 130.1 (DMT), 128.2 (DMT), 127.9 (DMT), 126.9 (DMT), 117.4 (CN), 115.6 (DMT), 113.1 (C5), 86.4 (DMT), 85.3 (C4´), 85.0 (C1´), 74.0 (DMT), 73.7 (DMT), 73.6 (C3´), 63.5 (C5´), 58.1 (OCH_2), 55.2 (OCH_3), 45.3 (2 × ^{i}Pr-CH), 40.5 (C2´), 24.6 (2 × ^{i}Pr), 24.5 (2 × ^{i}Pr), 24.4 (CH_2CN), 13.4 (Im-CH_3); ^{31}P NMR (162 MHz, $CDCl_3$): δ/ppm: 148.9.

1´-(4-Methylimidazol-1-yl)-2´-desoxy-3´-((2-cyanoethyl)-*N,N*-diisopropylphosphoramidit)-5´-*O*-4,4´-dimethoxytriphenylmethyl-β-D-ribofuranose (**4b**)

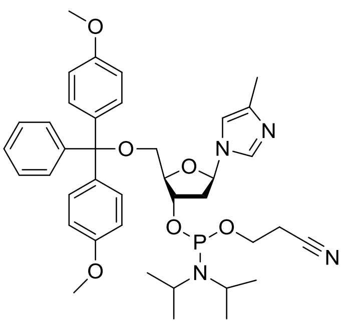

4b

Colourless oil; yield (193 mg, 69%) ^1H NMR (400 MHz, $CDCl_3$) δ/ppm: 7.53 (s, 1H, H2), 7.41 (m, 2H, DMT), 7.27 (m, 7H, DMT), 6.81 (m, 4H, DMT), 6.75 (s, 1H, H5), 5.94 (pt, 1H, H1´), 4.60 (m, 1H, H3´), 4.20 (m, 1H, H4´), 3.79 (s, 6H, OCH_3), 3.63 (m, 4H, CH_2), 3.59 (m, 2H, ^{i}Pr-CH), 3.28 (m, 2H, H5´/H5´´), 2.44 (m, 2H, H2´/H2´´), 2.15 (m, 3H, CH_3), 1.17 (m, 12H, ^{i}Pr); ^{13}C NMR (101 MHz, $CDCl_3$) δ/ppm: 158.5 (DMT), 144.5 (DMT), 135.7 (C4), 130.1 (DMT), 130.0 (DMT), 128.2 (DMT), 127.8 (DMT), 117.3 (CN), 113.3 (DMT), 113.1 (C5), 86.4 (DMT), 85.9 (C4´), 85.1 (C1´), 74.3 (DMT), 74.1 (DMT), 73.6 (C3´), 63.6 (C5´), 58.2 (OCH_2), 55.2 (OCH_3), 43.2 (^{i}Pr-CH), 43.1 (^{i}Pr-CH), 40.9 (C2´), 24.6 (^{i}Pr), 24.5 (^{i}Pr), 24.4 (2 × ^{i}Pr, *C*H_2CN), 13.6 (CH_3); ^{31}P NMR (162 MHz, $CDCl_3$): δ/ppm: 148.8; 148.7.

Molecular masses of the oligonucleotides as determined by mass spectrometry.

Sequence (artificial nucleosides marked in bold)	Mass (calcd.) / Da	Mass (found) / Da
5'-d(TTT GTT TGT TTG **2**TT GTT TTT TTT TT)	7904	7911
5'-d(AAA CAA ACA AAC **2**AA CAA AAA AAA AA)	7933	7940
5'-d(TTT GTT TGT TTG **4**TT GTT TTT TTT TT)	7904	7907
5'-d(AAA CAA ACA AAC **4**AA CAA AAA AAA AA)	7933	7932
5'-d(TTT GTT TGT TTG **22**T GTT TTT TTT TT)	7860	7860
5'-d(AAA CAA ACA AAC **22**A CAA AAA AAA AA)	7880	7888
5'-d(TTT GTT TGT TTG **44**T GTT TTT TTT TT)	7860	7868
5'-d(AAA CAA ACA AAC **44**A CAA AAA AAA AA)	7880	7889

Reference

[1] V. Rolland, M. Kotera, J. Lhomme, *Synth. Commun.*, **1997**, *27*, 3505-3511.

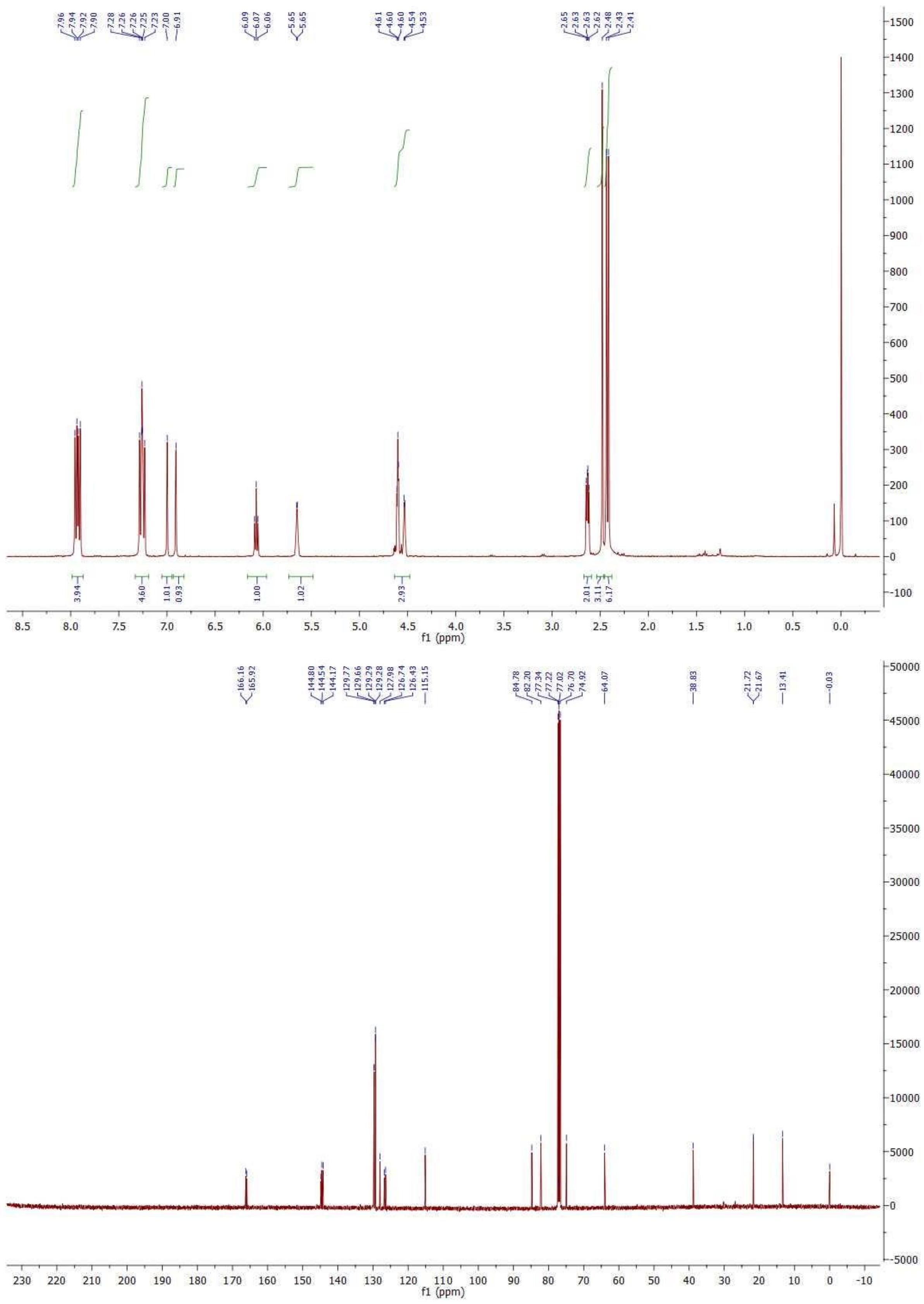

Figure S3: ^{1}H and ^{13}C NMR spectrum ($CDCl_3$) of compound **1a.**

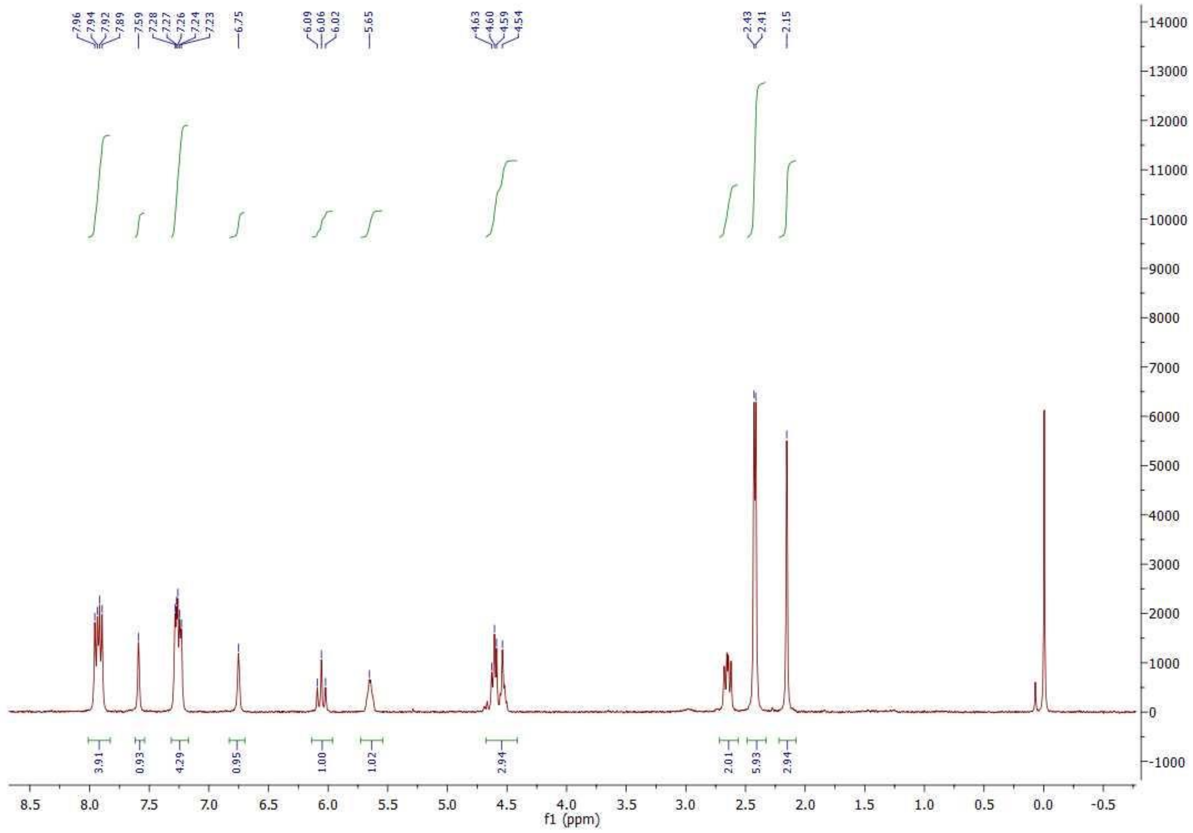

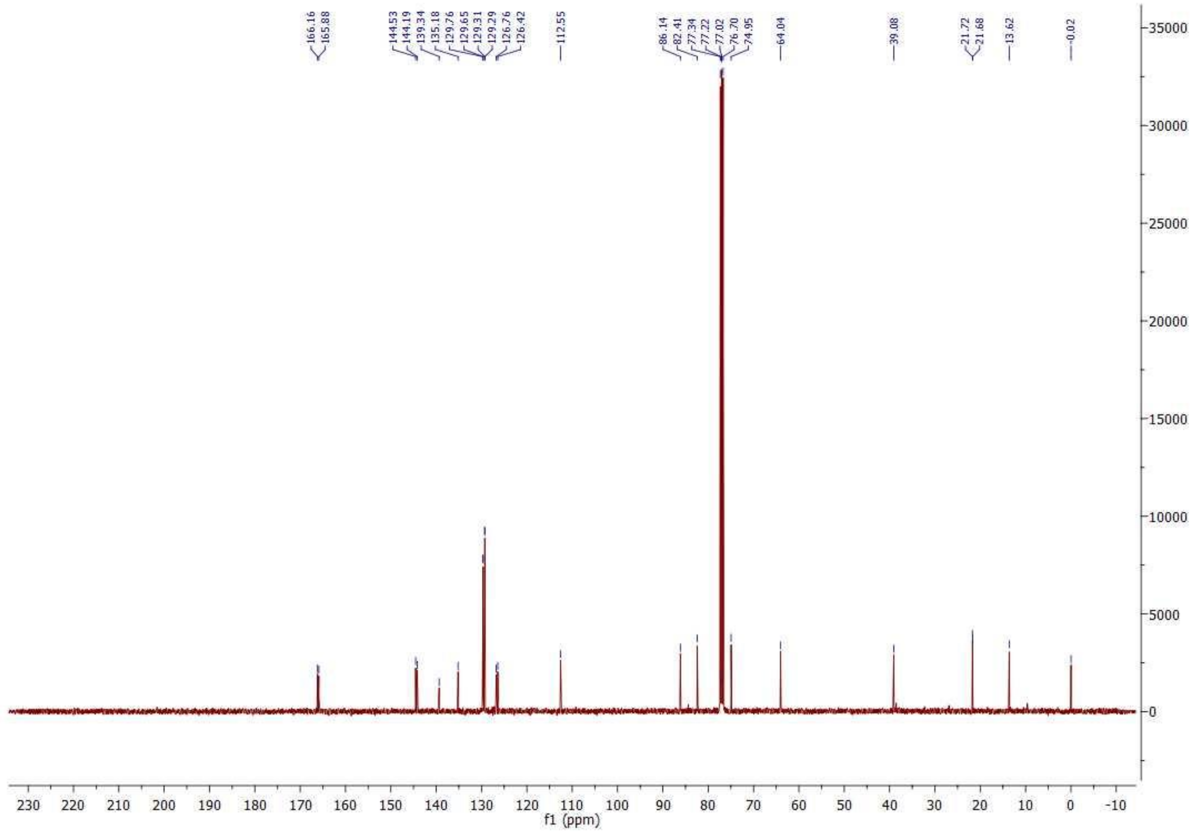

Figure S4: ^{1}H and ^{13}C NMR spectrum ($CDCl_3$) of compound **1b.**

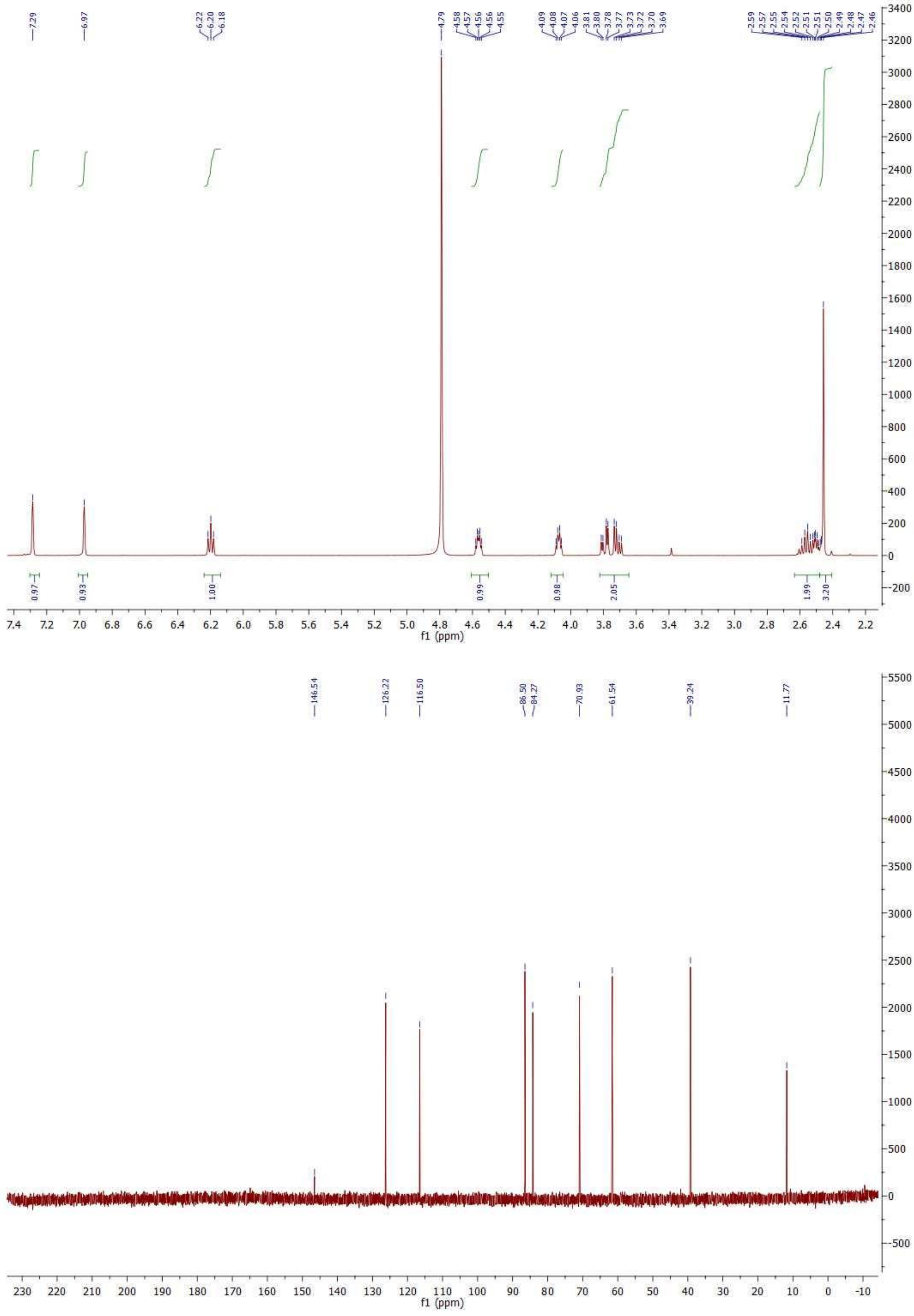

Figure S5: ^{1}H and ^{13}C NMR spectrum (D_2O, pD 8.6) of compound **2a.**

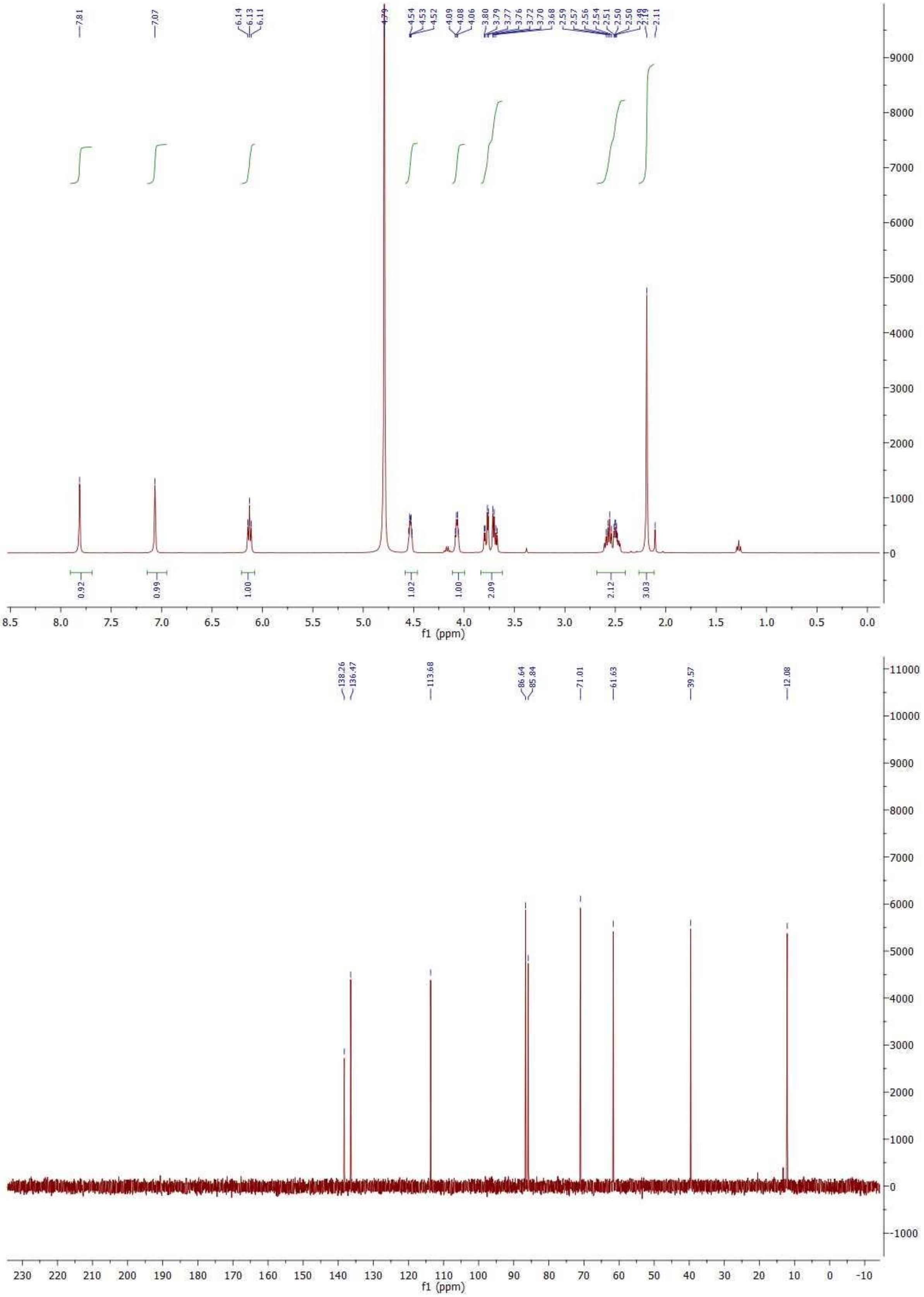

Figure S6: ^{1}H and ^{13}C NMR spectrum (D_2O, pD 8.8) of compound **2b.**

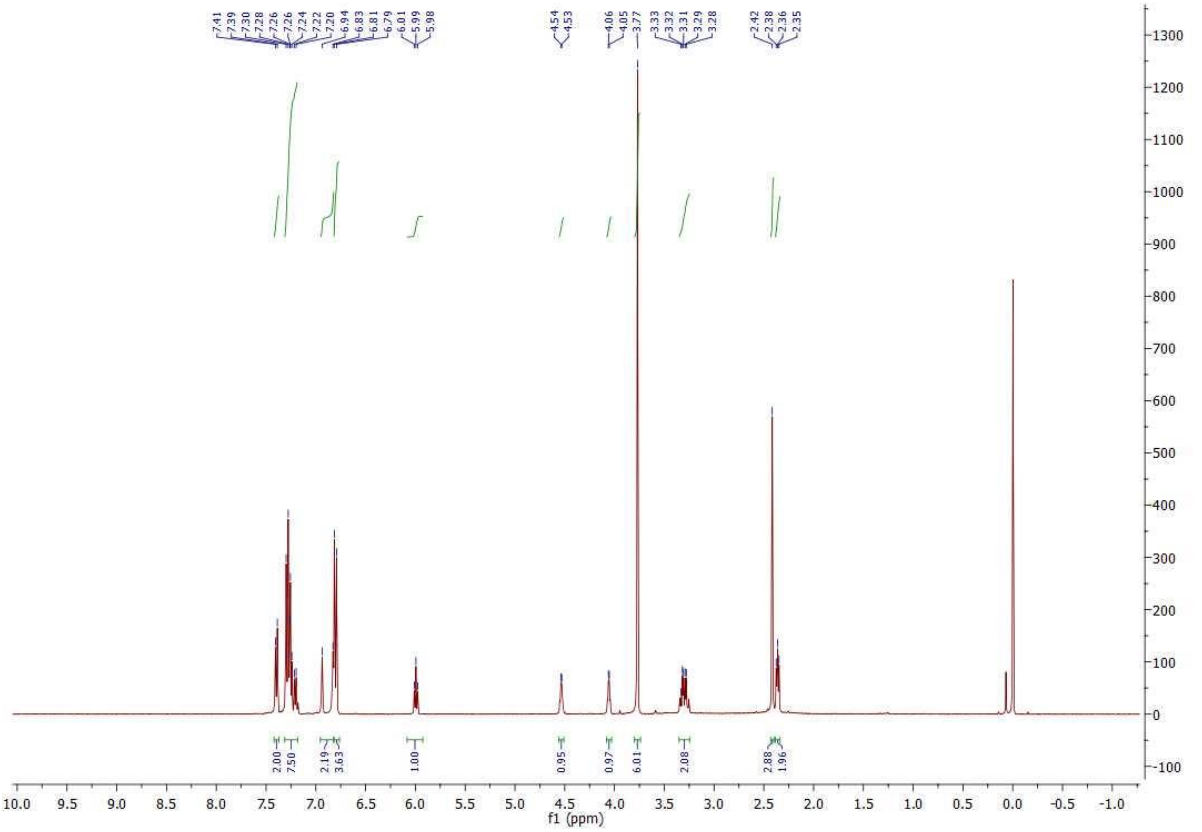

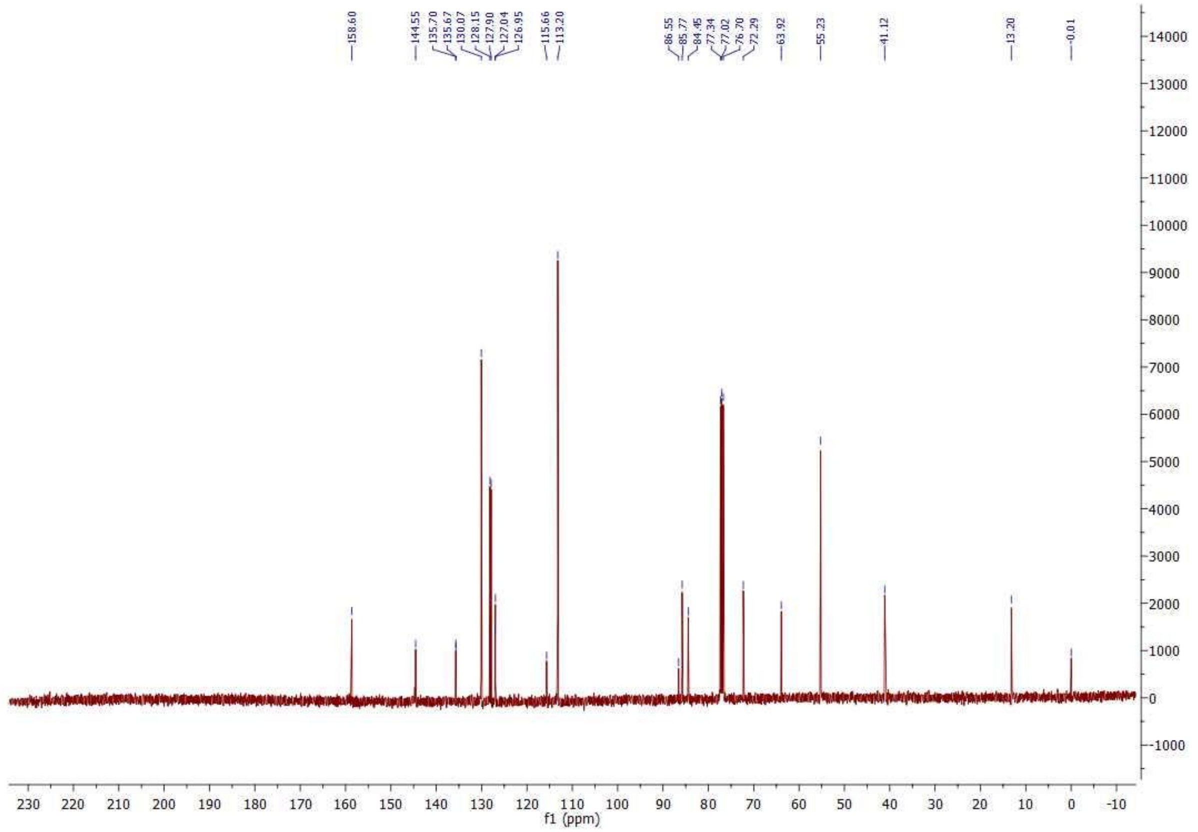

Figure S7: ^{1}H and ^{13}C NMR spectrum ($CDCl_3$) of compound **3a.**

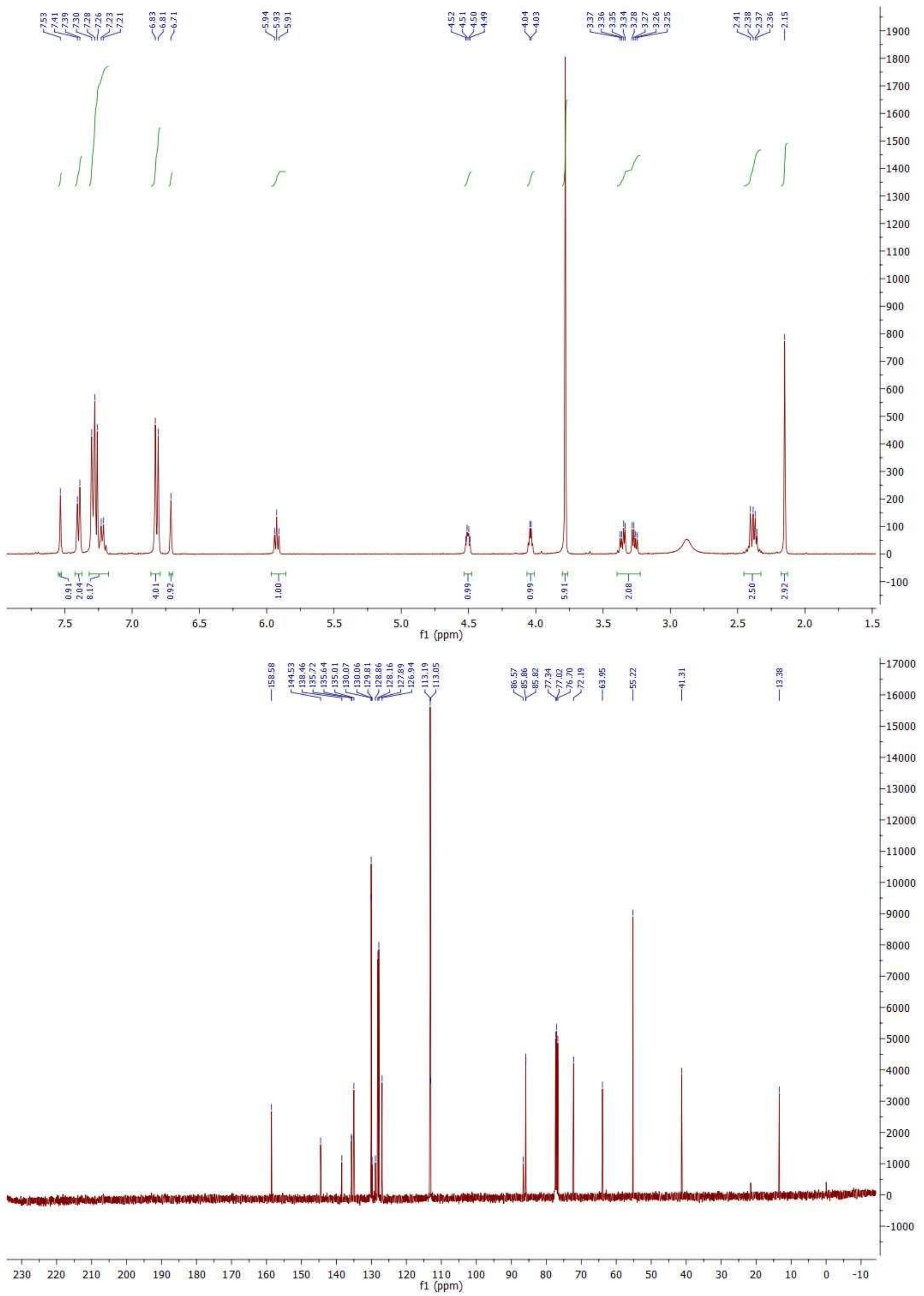

Figure S8: ^{1}H and ^{13}C NMR spectrum ($CDCl_3$) of compound **3b**.

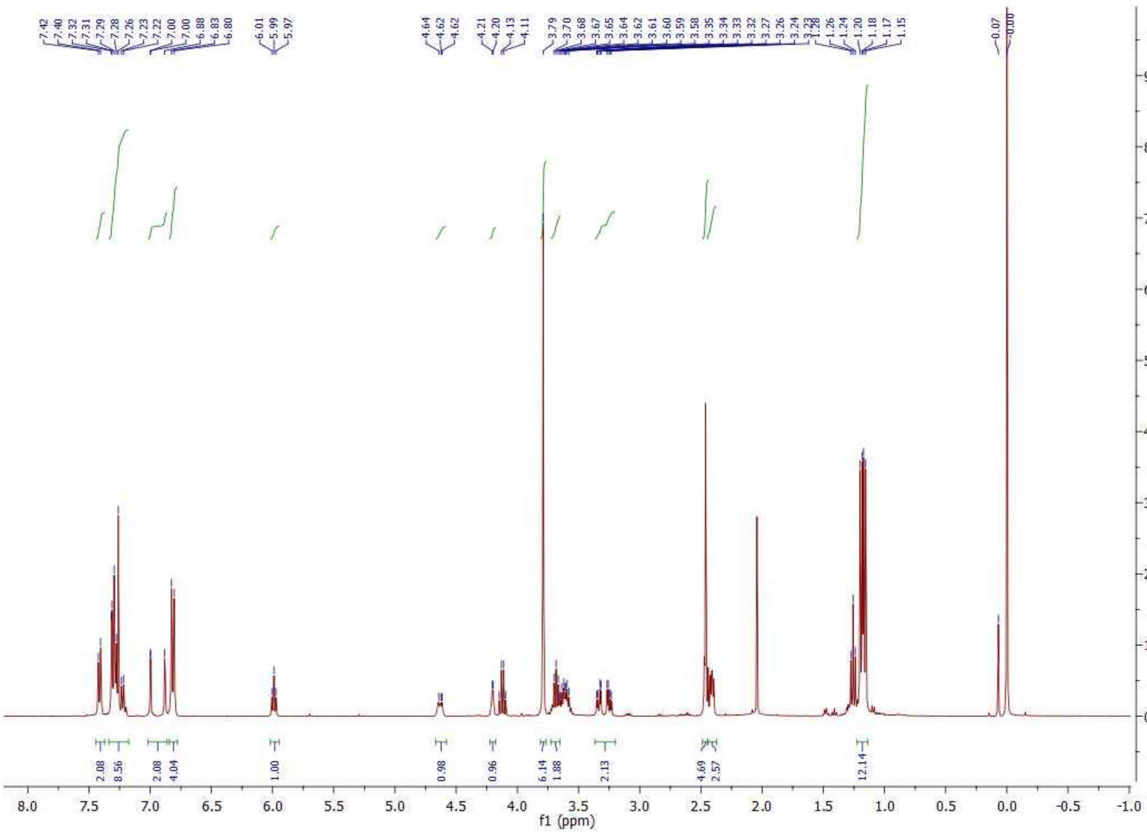

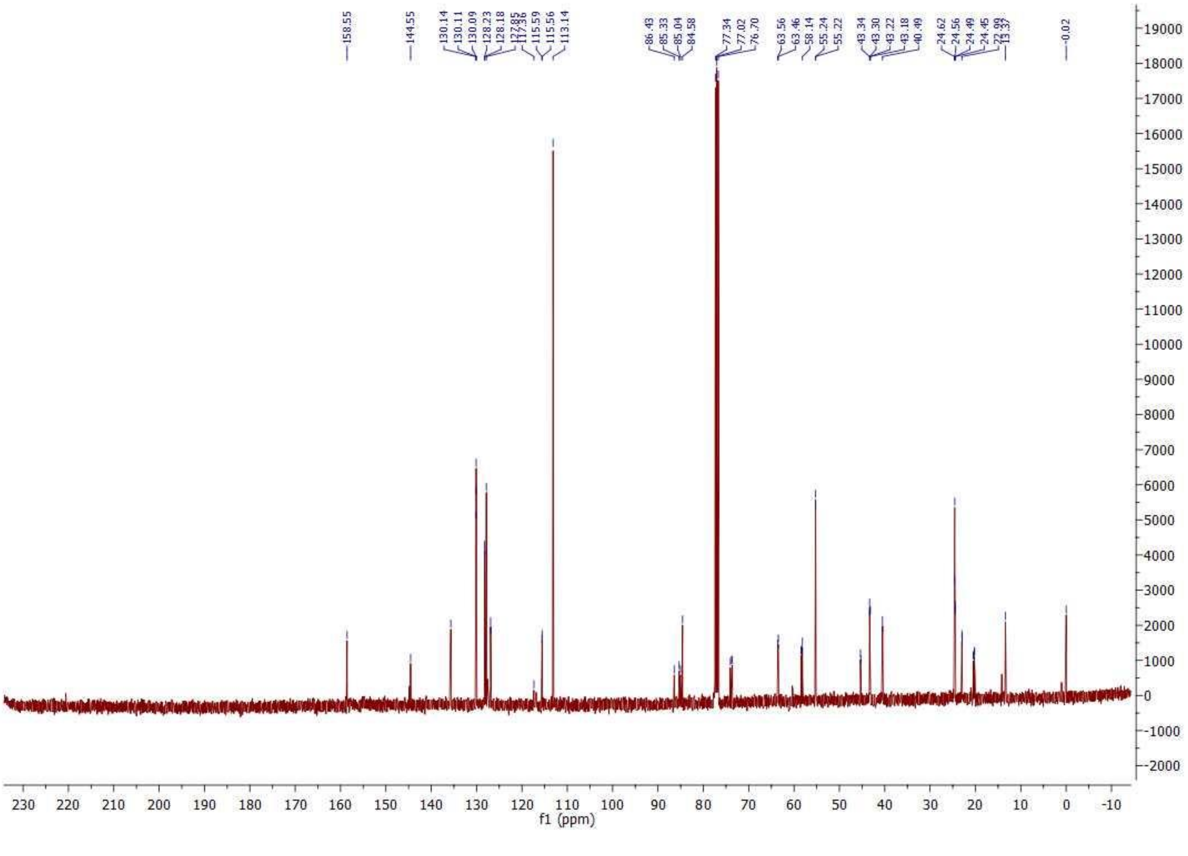

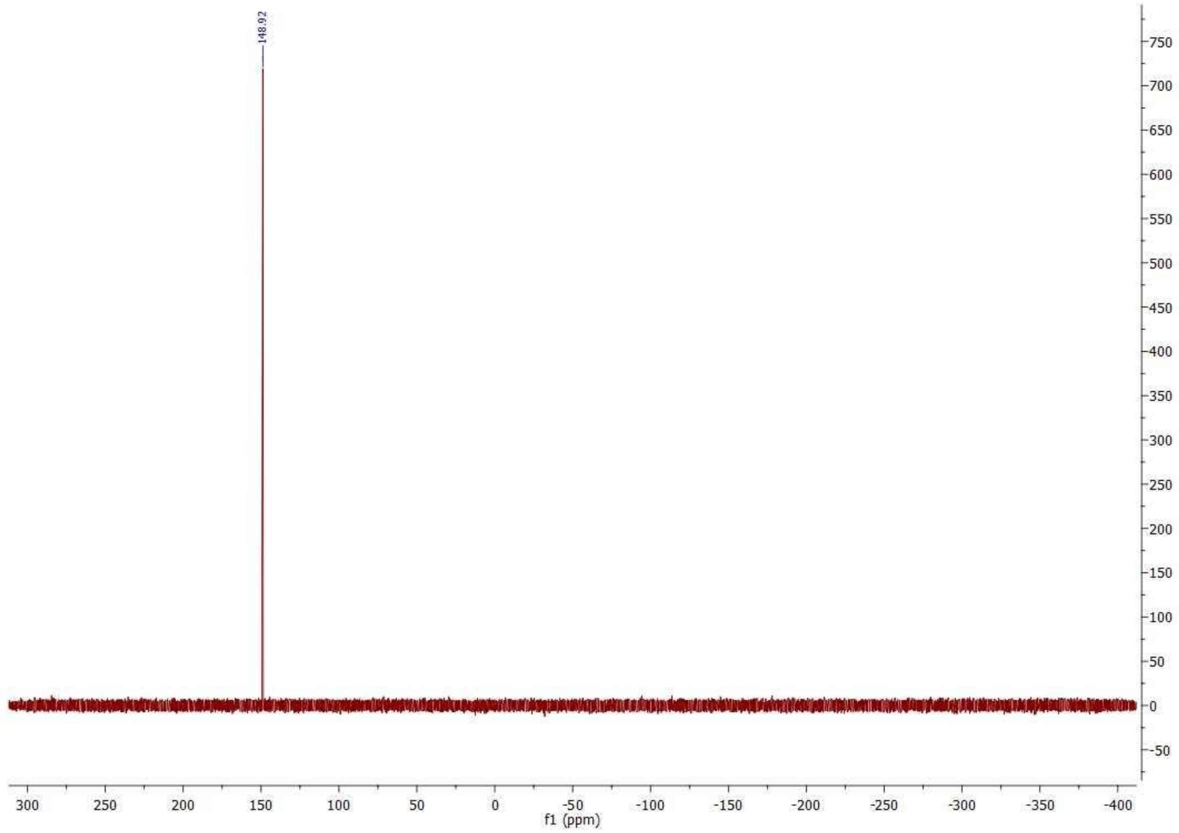

Figure S9: ^{1}H, ^{13}C and ^{31}P NMR spectrum ($CDCl_3$) of compound **4a.**

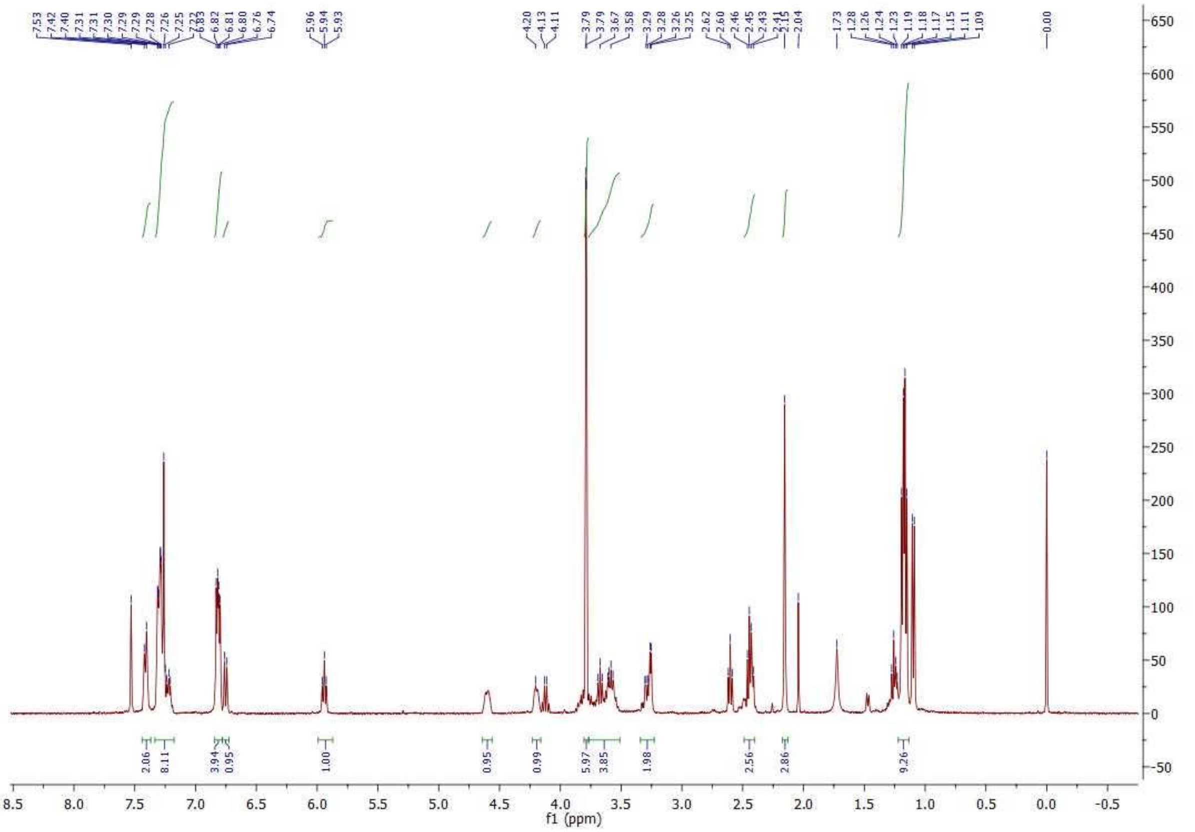

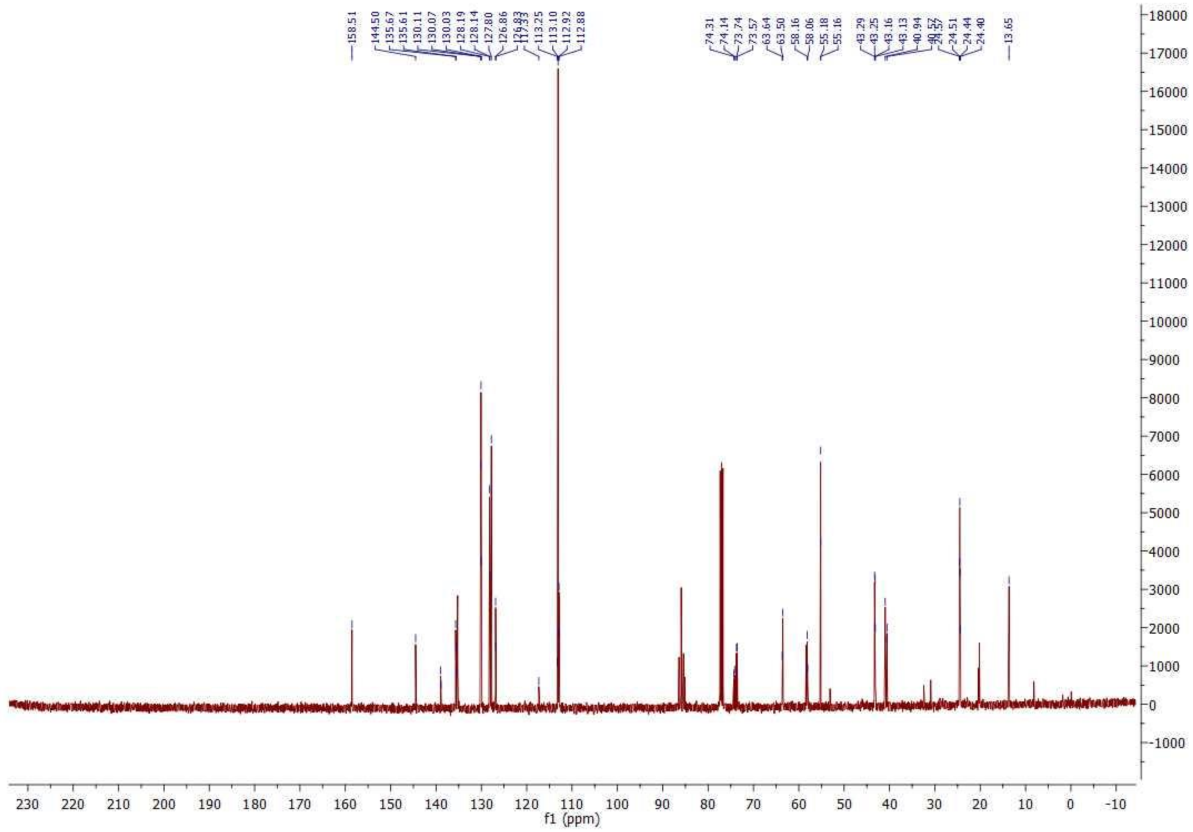

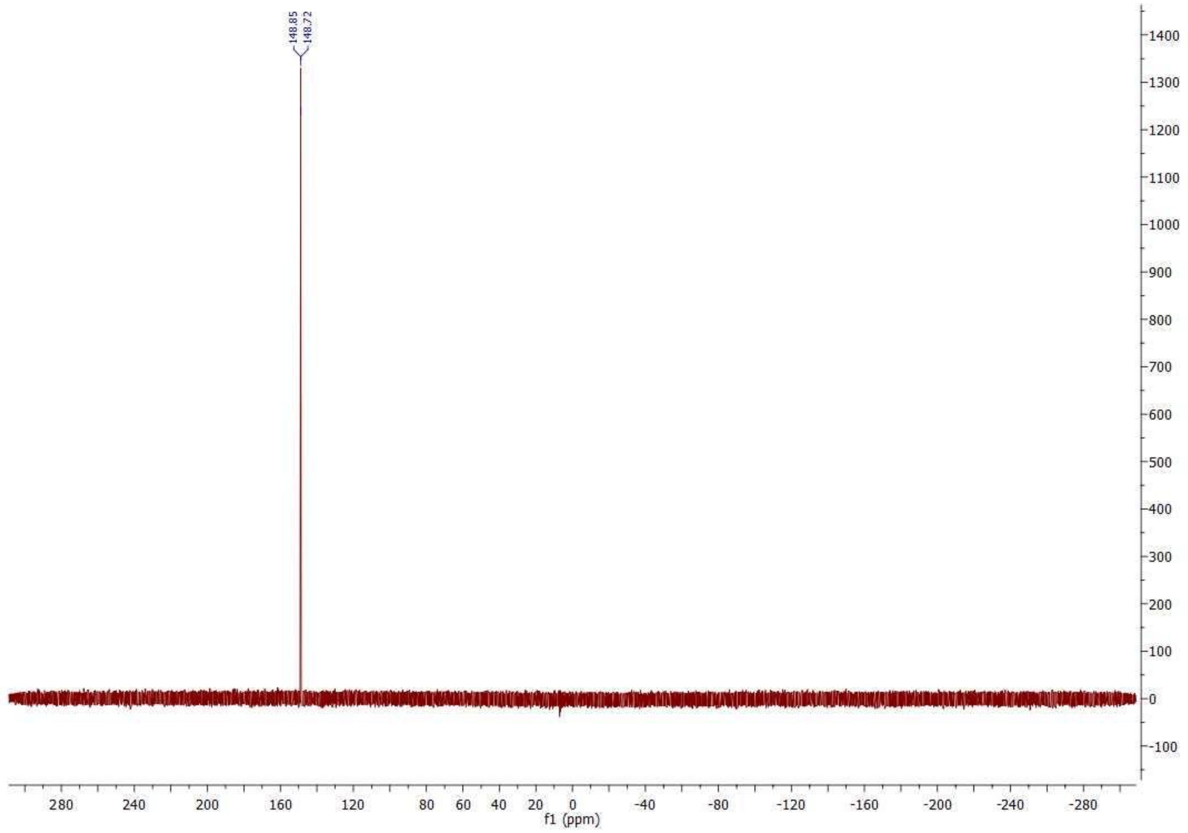

Figure S10: ^{1}H, ^{13}C and ^{31}P NMR spectrum ($CDCl_3$) of compound **4b.**
